# Hybrid type 1 effectiveness-implementation studies: why and how to do them

**DOI:** 10.3389/frhs.2026.1678257

**Published:** 2026-03-05

**Authors:** Jure Baloh, Sara J. Landes, Jeffrey L. Smith, Geoffrey M. Curran

**Affiliations:** 1 Department of Health Policy and Management, University of Arkansas for Medical Sciences, Little Rock, AR, United States; 2 Department of Psychiatry, University of Arkansas for Medical Sciences, Little Rock, AR, United States; 3 Behavioral Health Quality Enhancement Research Initiative (QUERI), Central Arkansas Veterans Healthcare System, Little Rock, AR, United States; 4 Department of Pharmacy Practice, University of Arkansas for Medical Sciences, Little Rock, AR, United States

**Keywords:** hybrid effectiveness-implementation, hybrid type 1, implementation, methodological guidance, resources

## Abstract

Effectiveness-implementation hybrid type 1 studies primarily investigate the effectiveness of an intervention and have a secondary focus on exploring implementation-related factors. Integrating implementation aims into intervention effectiveness studies can improve the speed, quantity, and quality of intervention implementation, sustainment, and scale in routine practice, and thereby maximize the impact on population health. This article provides guidance for designing and conducting the implementation aims of effectiveness-implementation hybrid type 1 studies, summarizing past thinking and advancing new considerations for these approaches. The authors argue that hybrid type 1 approaches are suitable for most types of intervention effectiveness research (e.g., efficacy trials, comparative-effectiveness research, observational studies), for different kinds of interventions (e.g., treatment, screening, prevention), and in a broad range of settings (e.g., healthcare, public health, community, schools). The article offers methodological guidance for designing the implementation aims of hybrid type 1 studies, structured around three goals: (1) explain intervention implementation in the effectiveness trial, (2) explore stakeholder perceptions to inform future implementation research, and (3) examine stakeholder perceptions to inform the effectiveness trial. Each of these goals offers a distinct set of research questions and design considerations (e.g., timing, sampling, data collection). Finally, the authors provide some tools and resources for planning and designing hybrid type 1 studies.

## Introduction

The traditional clinical research pipeline promotes a staged approach to developing evidence-based interventions (EBI) by first ensuring they work under ideal or near-optimal conditions (i.e., performs well first in highly-controlled efficacy trials and then effectiveness studies) without taking into consideration how they might ultimately be implemented in real-world settings (1, 2). As a result, substantial time may pass (3, 4) before an EBI is integrated into routine practice in clinical or community settings.

Determinants that influence why EBIs are or are not used in routine practice may include contextual factors (e.g., leadership support, organizational culture/climate, lack of funding, policy), characteristics of EBI deliverers and recipients (e.g., provider skills/knowledge in using EBI, resistance by end-users such as patients or community members), and characteristics of the EBI itself (e.g., complexity, resource requirements, relative advantage over current practice) (5, 6). Knowledge of implementation factors is critical to inform subsequent selection and tailoring of implementation strategies to support uptake of EBI in routine practice (7, 8). While effectiveness studies explored some of these factors in the past (9, 10), predating the “hybrid” terminology, many studies focused on factors more closely aligned with effectiveness (e.g., fidelity, reach) (11) and a broader range of implementation factors are not examined routinely despite availability of established methods to systematically identify barriers and facilitators that influence intervention implementation. Arguably, neglecting to explore these factors represents a lost opportunity to maximize what can be learned in intervention effectiveness trials to better inform subsequent efforts to implement, sustain, and scale EBIs in routine practice.

*Implementation science* has been defined as the study of the use of strategies to adopt and integrate EBIs into clinical and community settings (taking into account barriers and facilitators) to improve individual outcomes and benefit population health (12). Over a decade ago, implementation scientists proposed the use of *hybrid effectiveness-implementation studies* (1, 13, 14) to potentially address the above-noted concerns, as they promote application/examination of *both* effectiveness and implementation research questions within a given study. The proposition is that giving greater attention to assessing implementation processes and outcomes in hybrid effectiveness-implementation studies (while novel or adapted interventions are being evaluated) may enhance learning, improve research efficiency, and accelerate the pace of moving EBIs into routine practice.

Curran and colleagues (13) proposed three “ideal types” of hybrid effectiveness-implementation studies, each with differing levels of emphasis on research questions directed at the effectiveness of an intervention or implementation strategies supporting it. Type 1 studies focus mostly on the intervention; type 3 focus mostly on implementation strategies; and type 2 applies a more equal focus on the intervention and implementation strategies simultaneously. While the three “ideal types” suggest a crisp categorization, there remain critical gray areas, particularly when interventions are complex and effectiveness studies move away from highly controlled conditions and randomized controlled trials (RCT) designs towards more pragmatic, real-world evaluations. As even noted by Curran and colleagues (14), the types are “a useful shorthand for communicating the intent of the researchers but can fail to capture nuances of the study when a discrete choice of type is necessitated but not necessarily perfectly aligned.” Since their introduction in 2012, hybrid effectiveness-implementation studies have become widely used. In recent years, many authors have contributed new thinking and guidance to the use of hybrid studies (1, 14–18). The current manuscript adds to the growing literature on best practices for designing and using hybrid studies and is intended to assist researchers planning to evaluate the effectiveness of an intervention in determining whether a hybrid type 1 is appropriate for their work and, if so, guidance for planning and conducting implementation aims in such studies.

Of note, the original manuscript on hybrid studies focused on trial designs and was situated explicitly in the domains of health and healthcare delivery. Since then, the literature on hybrid studies, and indeed across the field of implementation science as a whole, has embraced use of these approaches and methods outside traditional clinical trial designs, across different intervention types, and in settings beyond health and healthcare (14, 19). The target audience for the current manuscript includes researchers working in diverse settings (e.g., healthcare, public health, community, schools), studying a variety of interventions (e.g., screening, prevention, treatment, recovery/support), and using a broad range of study designs (e.g., experiments, quasi-experiments, observational). While this manuscript continues to draw thinking and examples from RCTs and implementation in healthcare contexts, we try to include other examples, and we encourage wide application of the guidance provided herein.

In summary, hybrid type 1 studies have the potential to accelerate and enhance the uptake of EBI into routine practice by investigating the questions of “implementability” earlier in the development pipeline, during the stages of EBI development and testing. While hybrid type 1 approaches are increasingly common, strong potential remains for both increasing their use further and expanding the range of questions and methods employed to maximize what we learn from such studies. This article seeks to close this gap, and our primary target audience are effectiveness researchers and their teams who may be unsure whether to use a hybrid type 1 approach or not, or how to best integrate implementation aims with their effectiveness study. We first provide a brief introduction to implementation science and hybrid type 1 studies, summarize some considerations for when hybrid type 1s are useful (or not), and then articulate three distinct goals of implementation aims in these studies, along with describing key methodological considerations for each goal. We conclude by providing a hybrid type 1 planning tool and an annotated bibliography of type 1 studies to further help researchers design such studies.

### Basic introduction to implementation science concepts and terminology

As defined above, the goal of implementation science is to move EBIs into routine practice. Following Curran's (20) examples and terminology, we describe key concepts used in implementation science, which are particularly important for maintaining clarity in hybrid studies. At the center of implementation studies are evidence-based interventions (EBI), such as practices, programs, pills, and policies (2), with known or emerging evidence of effectiveness (i.e., impact on health outcomes). Some refer to them as the “object” (21) or simply “the thing” (20) being implemented. Efficacy/effectiveness research studies investigate whether “the thing works”—does the EBI improve health behaviors or outcomes, either in ideal conditions (efficacy) or more pragmatic, real-world conditions (effectiveness). While RCTs are considered the gold standard in healthcare and many other fields, other experimental and observational designs can contribute to the evidence base (22). Implementation science builds on and extends the research on EBIs by introducing implementation strategies, which are implementation interventions (e.g., conduct educational meetings, use clinical reminders, provide technical assistance) designed to help with adoption, implementation, and sustainment of EBIs in routine practice settings. Implementation research frequently investigates the impact of implementation strategies on implementation outcomes (e.g., reach, fidelity; see below); in a broader sense, implementation research also includes other types of studies investigating implementation questions (e.g., observational studies, simulation) and also includes what some call *implementation preparation* (e.g., documenting knowledge-practice gaps, assessing barriers and facilitators) (23, 24), which are essential steps in the process of selecting and designing practical implementation strategies before testing them. For implementation research, it is also important to distinguish between implementation determinants (barriers and facilitators), which are the factors influencing EBI implementation at multiple levels (e.g., patient/community member, provider, organization, community), and implementation outcomes, which indicate the extent (how much) and quality (how well) of intervention implementation (e.g., intervention adoption, reach, fidelity) and are thus necessary preconditions and antecedents of intervention effectiveness (20, 25, 26).

### Brief overview of hybrid type 1 effectiveness-implementation studies

Hybrid type 1 studies primarily investigate the effectiveness of an intervention and have a secondary focus on exploring implementation-related factors. The majority of published hybrid type 1 studies have used a person-level randomized RCT design for evaluating the intervention, augmented by an implementation-focused process evaluation of some kind (27). These process evaluations generally focus on the relative “implementability” (e.g., utility, acceptability, feasibility) of the intervention (from deliverer and recipient perspectives) and an assessment of the implementation contexts where the intervention was delivered during the study. The evaluation of implementation contexts often assesses barriers and facilitators to implementation during the study based on perspectives of people who participated in delivering the intervention and those who received it. Further, many hybrid type 1 studies also explore participants’ perspectives on what might be needed to support “real world” implementation in the future, such as how the intervention might benefit from adaptation to increase uptake/engagement, and what types of implementation strategies could be developed, used, and tested in future research. The majority of published hybrid type 1 studies to date have used an implementation framework of some kind (usually an implementation evaluation and/or determinant framework) (25, 28) to guide data collection and analysis. Qualitative methods are frequently used, and mixed method (qualitative-quantitative) approaches are growing.

The literature in recent years has seen hybrid type 1 studies applied in novel ways. For example, some investigators have expanded the scope to include “naïve” sites, meaning those not involved in the intervention effectiveness study and representing the kind of sites where the intervention would be implemented in the future (29, 30). In other novel applications, some investigators have conducted small-scale hybrid type 1 studies when pilot testing interventions or conducting early tests of intervention efficacy. Other investigators have added on an implementation-focused process evaluation to an already funded clinical trial that was not previously conceived as a hybrid type 1. The new guidance in this manuscript draws from and extends upon these innovations.

### When is a type 1 study more or less useful?

There has been debate in the field about when and under what circumstances hybrid studies generally, and hybrid type 1 studies specifically, are most “called for/useful” or not. Here we summarize some of our previous writing and offer new thoughts on this issue.

A hybrid type 1 study is perhaps most useful when intervention effectiveness data are limited or inconclusive, so an intensive focus on implementation strategies (e.g., hybrid type 2 or 3) is likely premature (1). Common situations here include when an intervention is newly developed (e.g., injectable treatment that replaces a pill), when an intervention is significantly adapted for a new population or setting (e.g., obesity prevention intervention adapted for delivery in schools), or when interventions are bundled in novel ways that warrant testing their collective effectiveness (e.g., bundling medication and psychotherapy). Typically, a hybrid type 1 study is most useful when these interventions have been previously shown to be efficacious or their components/prior versions were shown to be efficacious (i.e., in smaller, highly controlled studies) and/or effective (i.e., in larger, more real-world studies), and patient safety is not a major concern.

Hybrid type 1 studies may be less appropriate in situations when patient/recipient safety is not yet well-understood, or when pursuing regulatory approval (e.g., U.S. Food and Drug Administration approval), and thus the bulk of study resources are understandably dedicated to intervention efficacy/effectiveness. In these cases, it might be considered too soon for a hybrid type 1 study. Further, there are situations where an intervention effectiveness study with an implementation-focused process evaluation would likely yield limited benefit for the effort. Examples include studies of interventions that are very similar to the treatment as usual condition (e.g., new pill vs. current pill) or where there already exists a well-developed understanding of the implementation questions associated with the intervention and the context where it will be implemented.

We acknowledge that many intervention effectiveness studies already consider and assess factors that influence intervention implementation. Furthermore, all intervention effectiveness trials use strategies to ensure rigor and support the delivery of the intervention, such as conducting educational meetings, paying clinics and interventionists, and conducting frequent fidelity checks (and intervening when fidelity wavers). These strategies can be considered implementation strategies—and some may indeed serve as practical/real world implementation strategies to include in future efforts to implement the intervention (e.g., training). However, some strategies used in effectiveness trials can be resource-intensive and may not be feasible for supporting wide-spread adoption of the intervention outside of research (16). In hybrid type 1 studies, a robust implementation-focused process evaluation (as mentioned above) allows the researcher to explore and identify such feasibility concerns to inform adaptations to implementation strategy for testing in future intervention implementation efforts.

Some (31) have argued that for *clinical* intervention *effectiveness trials* in particular, i.e., those evaluating interventions with previously demonstrated efficacy (and/or prior effectiveness results with highly similar interventions), hybrid type 1 approaches should be the default. At the moment, most major extramural funders in the US and elsewhere have yet to set such a default; however, many requests for applications recognize and recommend consideration of hybrid designs, including hybrid type 1 (32–34). Further, authors of this manuscript (SJL, GMC) are currently involved in multiple hybrid type 1 studies where the PIs were recommended by reviewers or funders to add an implementation-focused process evaluation to their non-hybrid effectiveness study proposal. Given the continued interest in and proliferation of hybrid type 1 studies, it is important for the field to provide more guidance to those considering and developing such studies.

## Methods

In this section, we provide guidance and recommendations for conceptualizing and designing the implementation aims of hybrid type 1 studies. As relevant, we also discuss considerations for the intervention effectiveness aim(s), but assume the reader has prior knowledge and understanding of effectiveness studies/trials, related design considerations, and the associated norms from their respective fields. Furthermore, the details of the methods used for implementation aims (e.g., how to conduct qualitative interviews, how to conduct mixed-methods analyses) are outside the scope of this paper and we refer the reader to the literature on those methods (35–40) and additional resources at the end of this document.

Here, we identify three broad goals (or purposes) of the implementation aims in hybrid type 1 studies:

- Goal 1: Explain intervention implementation in the effectiveness trial
- Goal 2: Explore stakeholder perceptions to inform future implementation research
- Goal 3: Examine stakeholder perceptions to inform the effectiveness trial

We refer to them as *goals 1, 2, and 3* throughout the paper. Each of these goals is associated with a set of design considerations in relation to the intervention effectiveness study (e.g., timing, sampling, data collection). These design considerations are depicted in Table 1 and described in more detail in the following paragraphs, which are organized around the three goals. It is important to note that the goals are not mutually exclusive (studies can combine two or three of these goals), and new goals may be articulated or developed in the future. In other words, we see these goals as a menu of options to consider when planning a hybrid type 1 study. Of note, we are not recommending that people lengthen the name of hybrid type 1 studies (e.g., calling them “hybrid type 1, goal 2”).

**Table 1:** Design considerations for the three goals of implementation aims in hybrid type 1 studies.

Design considerations	Goal 1: Explain intervention implementation in the effectiveness trial	Goal 2: Explore stakeholder perceptions to inform future implementation	Goal 3: Examine stakeholder perceptions to inform the effectiveness trial
Types of implementation aims/questions (examples)	• Evaluate implementation processes and outcomes in the effectiveness trial • Explore heterogeneity (subgroup analyses) of intervention outcomes	• Document implementation determinants • Explore how to support intervention implementation in routine practice • Identify need for intervention adaptation	• Determine/refine intervention design, adaptation, or bundles before trialing • Refine evaluation design (e.g., outcomes) • Inform strategies/support for the trial
Study design (aims sequence and interaction)	• Effectiveness aims (trial) mostly precede implementation aims (observational) • Minimal interaction between aims and minimal threat to trial integrity	• Effectiveness aims (trial) precede implementation aims • No interaction between aims and no threat to trial integrity	• Implementation aims (observational) precede effectiveness aims (trial) • Large interaction and risk between aims; however, can improve trial design
Evaluation	• Implementation-focused process evaluation to explain intervention implementation, heterogeneity, etc. • Quant or mixed methods (QUANT-qual)	• Implementation-focused process evaluation to explore implementation determinants and future recommendations • Qual or mixed methods (QUAL-quant)	• Intervention- and implementation-focused formative evaluation to examine innovation and implementation factors • Qual or mixed methods (QUAL-quant)
Theories, models, and frameworks	• Outcome frameworks (e.g., RE-AIM) • Determinant frameworks (e.g., CFIR) • Classic theories (e.g., agency theory)	• Determinant frameworks (e.g., CFIR) • Outcome frameworks (e.g., RE-AIM, particularly for *perceptions* of outcomes)	• Determinant frameworks (e.g., CFIR, i-PARIHS, TDF) • Process models (e.g., ADAPT-ITT)
Implementation sample	• Intervention deliverers (e.g., trained providers, teachers, research staff) • Intervention recipients (e.g., clients, patients, students, community members)	• Intervention deliverers (e.g., providers) • Intervention recipients (e.g., clients) • Other relevant groups (e.g., other staff, leadership, purveyors, payors)	• *Targeted* intervention recipients • *Planned* intervention deliverers • Other relevant groups (e.g., leaders, payors, purveyors, community partners)
Implementation data collection and types	• Measures: Implementation processes and outcomes; some determinants • Data types/sources: Trial data+administrative data (e.g., EHR, study logs) + other quantitative (e.g., surveys) and qualitative data (e.g., interviews)	• Measures: Implementation determinants; some outcomes (e.g., acceptability); future recommendations (e.g., strategies) • Data types/sources: Qualitative data (e.g., interviews, focus groups) and quantitative data (e.g., surveys)	• Measures: implementation and innovation determinants, some outcomes (e.g., acceptability) • Data types/sources: Qualitative data (e.g., interviews, focus groups; usability testing), quantitative data (e.g., surveys)
Timing	• Design: Best designed before trial • Data collection: During and/or after trial • Analyses: Best after trial (trial integrity)	• Design: Before trial or post-hoc • Data collection: After intervention/trial • Analyses: After trial	• Design: Must be designed before trial • Data collection: Before intervention/trial • Analyses: Before intervention/trial
Resources and team expertise	• Low-medium resource intensity (planning, data collection, analysis) • D&I expert is more involved in design, execution, and interpretation	• Low resource intensity (minimal and low-risk data collection) • D&I expert to design and supervise, trained staff or postdoc can execute	• Medium-high resource intensity (time & expertise to plan and execute) • D&I expert integral member of the team

RE-AIM, Reach Effectiveness Adoption Implementation Maintenance framework; CFIR, Consolidated Framework for Implementation Research; i-PARIHS, integrated Promoting Action on Research Implementation in Health Services framework; TDF, Theoretical Domains Framework; ADAPT-ITT, Assessment, Decision, Adaptation, Production, Topical experts-integration, Training, and Testing model; EHR, Electronic Health Record.

### Goal 1: explain intervention implementation in the effectiveness trial

#### Purpose and research questions

The first goal primarily involves evaluation of intervention implementation processes and outcomes that occur in (during) the intervention effectiveness study, with the aim of understanding what happened in the trial and exploring relationships between variables of interest. The research questions can explore things such as treatment heterogeneity or health equity (Which subgroups of intervention recipients received the intervention, and/or benefited most/least from it, and why?), actual implementation of the intervention (Which providers delivered the intervention, how much, and with what fidelity? What was the cost or cost-effectiveness of the intervention?), and even implementation strategy-focused questions (How many and what type of providers attended training, or to what extent was fidelity obtained/maintained). Additionally, when effectiveness studies produce null results (e.g., intervention is not effective) or fail (e.g., low recruitment, variable intervention delivery), such implementation-focused process evaluations can serve as a “post-mortem” and help researchers identify the potential reasons for these outcomes (e.g., was poor implementation associated with the failure?), which can then inform future effectiveness work (e.g., intervention redesign, better support strategies).

#### Evaluation design and framework

To minimize the risk of contaminating the intervention effectiveness study (particularly if an RCT design is used) and other threats to its integrity, the team needs to consider how to integrate implementation aims in the overall study design. While the key risks mostly pertain to participant recruitment and data collection (which could influence their behavior in the study; see more on this below), it is equally important to stress that data should be analyzed after the trial procedures are complete, to avoid inadvertently biasing the research team and their behaviors (e.g., acting on knowledge of treatment heterogeneity), unless the study is explicitly designed to use these data, such as for sampling purposes (e.g., recruitment target by demographics) or tailoring variables in adaptive designs (e.g., change in treatment based on early outcomes).

At the most basic level, the team could use data generated in the effectiveness study to investigate implementation questions (e.g., explore intervention reach by participant demographics). In the parlance of clinical trialists, this is often called process data. Additionally, administrative data [e.g., study logs, electronic health records (EHR), school records] can be used to complement trial process data, without requiring any additional interaction with participants or providers. These approaches pose no additional risk to the integrity of the intervention effectiveness trial other than inadvertently influencing research team behavior. However, these data alone might allow for only a surface look at implementation processes.

Investigation of implementation questions can be enhanced by collecting additional primary data (e.g., surveys, interviews, observational data), and without too much budgetary or study timeline constraints. However, care needs to be taken on when and how these data are collected to reduce risk to the integrity of the trial/study (such as participants changing their behavior because of an interview question). To minimize such risk, implementation-focused data are typically collected after individuals (e.g., providers, patients, community members) are no longer directly involved with the intervention and its delivery in the trial. However, some data collection can be included earlier in the study, particularly if that improves scientific rigor (e.g., measuring provider intent to use intervention soon after training) or logistical feasibility (e.g., collecting implementation data during site visits, combining effectiveness and implementation questions on the same survey). In such instances, recruitment and data collection need to be designed carefully to minimize participant burden and ensure the intervention effectiveness trial is not adversely impacted.

Evaluation is typically designed as an implementation-focused process evaluation (27). Ideally, process evaluation is designed to accompany/extend the summative evaluation of the intervention (i.e., Does the intervention work?), which allows for more nuanced analyses of intervention outcomes (e.g., why and how it works). In addition to analyses of heterogeneity (subgroup analyses), process-focused evaluation can examine implementation outcomes to unpack whether the intervention was delivered as intended (e.g., adoption, reach, fidelity) and which factors were associated with these outcomes. While several of these factors are controlled in efficacy trials (e.g., patient eligibility criteria, providers are required to maintain a certain level of fidelity), effectiveness trials can allow a degree of variability, which can be explored analytically.

Many of these process-oriented variables are implementation outcomes, and thus evaluation frameworks (25) such as RE-AIM (41) and Proctor outcomes framework (26) are ideally suited for the analyses. Additionally, determinant frameworks such as CFIR (5) or EPIS (42) can guide systematic investigation of a broad range of factors (determinants) that can influence implementation, particularly at levels of inner context (e.g., leadership support, available resources) and individuals (e.g., self-efficacy, motivation, intention to use the intervention) within the settings involved in the trial. Several of the evaluation and determinant frameworks have recently also been applied in or adapted for investigation of health equity questions [e.g., RE-AIM (43), PRISM (44), CFIR (5)], and health equity-focused implementation frameworks (45, 46) now exist to offer more explicit guidance. Finally, many classic theories (theories originating outside implementation science) (25) can facilitate exploration of intervention and implementation mechanisms (e.g., Did the intervention change participant health behavior? Did the training strategy change provider knowledge?). For a more nuanced explanation and guidance on the types of implementation science frameworks, please see Nilsen's classic paper (22); tools such as T-CaST (47) can further assist selection of specific frameworks.

Implementation-focused process evaluations are frequently designed as mixed-methods evaluations, combining trial process data with additional implementation-focused quantitative and qualitative data (e.g., surveys, interviews, observations) to provide a fuller understanding of the implementation processes as they occurred in the intervention effectiveness study.

#### Sampling and data collection

With its focus on investigating what happened during the trial, purposive and convenience sampling approaches provide a good balance of rigor and feasibility. Individuals at the places involved in the study (e.g., patients/clients, providers, support staff, research staff) provide the most relevant information based on their direct experience with (and of) the study, and they are also relatively straightforward to identify and recruit. Snowball sampling can augment the above approaches by asking participants to identify other individuals or groups that played a critical (but potentially overlooked) role in the intervention or its implementation (e.g., family members of participants, technical support staff at the implementation site). Additionally, the principle of maximum variation is used in sampling to help record a full range of experiences (e.g., high and low adopters, health equity-focused subgroups), and we typically aim for saturation by role/type of person.

In addition to collecting intervention effectiveness study data (i.e., data for the effectiveness aims), the team needs to consider additional data collection to inform implementation aims, which we discuss here in approximately increasing levels of burden. At the minimum, implementation analyses can use study data to answer some implementation questions (e.g., exploring reach to targeted recipients by participant demographics). Other questions can be answered with the use of administrative data, including data from participating organizations (e.g., clinic/EHR records, school records) or the study itself (e.g., records of intervention training attendance). Such data require only minimal effort to collect (e.g., data use agreement with schools, notes taken/collected by study team), but can yield important insights, such as provider-level variation in intervention adoption and use. Next, any primary data collection (e.g., patient reported outcome measures) could be expanded in scope to include a few additional measures from intervention recipients (e.g., sociodemographic data), or practitioners delivering it (e.g., intention to use intervention, organizational readiness for change (48). Finally, more extensive primary data collection could be added, including observations by trained research staff (e.g., observation of hand hygiene on hospital units following implementation of educational posters), or additional qualitative or survey data collection (e.g., after teachers are trained on the intervention, measure training impact on their knowledge and intention to use the intervention). At all levels, researchers need to consider what data can be used to inform the questions of health equity—for example, factors such as age, sex, and race/ethnicity may be available in study or administrative data, while factors such as income, health literacy, or food insecurity would likely necessitate additional primary data collection.

#### Other considerations

Implementation evaluation associated with the first goal can be designed with different levels of intensity, and careful planning can yield a significant return on investment if collection of selected measures is designed into the conduct of the effectiveness study. Whereas some data can be collected after the fact and produce useful insights with limited resources (e.g., additional interviews, or secondary data analyses of administrative data), timely data collection during the study can sometimes offer most optimal results, such as surveying providers on intent to use the intervention soon after training to minimize recall bias, or directly observing intervention use while it is being deployed in the study. Including implementation researchers early in the design stage can facilitate data collection planning and execution and maximize what is learned in a given study.

### Goal 2: explore stakeholder perceptions to inform future implementation research

#### Purpose and research questions

The second goal primarily focuses on investigating perceptions of implementation determinants (i.e., barriers and facilitators) and gathering recommendations for addressing these determinants, with the goal of informing the selection and tailoring of implementation strategies for future implementation efforts. The implementation questions researchers can pursue may include “What are the implementation determinants for implementing intervention X in setting Y?”, or “How can we best support implementing X in setting Y under pragmatic, real-world conditions?”, or “What adaptation(s) to intervention X in setting Y may be needed for maximum real-world impact?”.

The determinants are typically the barriers and facilitators that individuals involved in the trial (e.g., patients, providers, study staff) experienced, but can also include *hypothetical* determinants that individuals think they would have experienced in real-world implementation. Information on hypothetical determinants can also be obtained from individuals that were not involved in the trial (see sampling below).

Additionally, researchers can obtain input and feedback on strategies that were used in the intervention effectiveness study to ensure that the intervention was provided (e.g., Did the participants find the training adequate and accessible?). These strategies could be used purposefully as practical implementation strategies in the future (e.g., conducting educational meetings, deploying clinical reminders, providing technical assistance) while other strategies may be less feasible in the real-world (e.g., recording and rating each session for fidelity). Obtaining practitioner feedback can inform their selection and/or adaptation for future implementation. Feedback could also lead to identification of new implementation strategies, which can be selected, tailored, and tested in future implementation studies.

Finally, while goals 1 and 2 are complementary and could be pursued in tandem on the same study, their focus is distinct and in some instances goal 2 represents an alternative to goal 1, particularly if resources are more constrained, risk to intervention trial is deemed too significant, or implementation aims are added ad-hoc (these issues are discussed in more detail below). However, it is worth noting that goal 2 introduces other risks, such as recall bias, loss of participant availability, or *post-hoc* rationalization, particularly if the time lag between intervention participation and implementation data collection is considerable.

#### Evaluation design and framework

The implementation aims associated with the second goal are conducted mostly separately from the intervention effectiveness study and typically follow the effectiveness aims, minimizing the potential risk or adverse impact on the effectiveness study. Data collection can be conducted after the individuals (e.g., providers, patients, community members) are directly involved with delivering or receiving the intervention and can occur sometime after the effectiveness study. The implementation aim can also be included as an add-on study or aim, after the intervention effectiveness study has already been designed, fielded, or even finished (e.g., if additional resources such as funding or personnel became available at a later point).

The implementation aim is typically designed as an implementation-focused process evaluation with the goal of obtaining key information to inform future implementation efforts, such as selection and tailoring of implementation strategies. Determinant frameworks (25), such as CFIR (5) or EPIS (42), are well-suited for this task, facilitating rigor and systematic investigation of a broad range of factors that can influence implementation. Additionally, evaluation frameworks [e.g., Proctor's Implementation Outcomes (26) or RE-AIM (41, 49) frameworks] could be used to augment exploration of individuals' perceptions of key implementation outcomes, such as perceptions of acceptability, appropriateness, and feasibility. While these *perceptions* can be considered as early implementation outcomes, they are also key determinants of downstream implementation *behaviors* (50). Of note, health equity-focused frameworks or adaptations of above frameworks can be used for a more nuanced and explicit study of participants' experiences and perceptions of health equity.

Finally, qualitative and mixed methods are best suited for collection and interpretation of relevant data (described in more detail below). Using only quantitative methods such as surveys would likely miss important nuances on key considerations and factors relevant to future implementation efforts, limiting their usefulness and appropriateness for this task.

#### Sampling and data collection

Similar to the first goal described earlier, sampling for the second goal is typically based around purposive, convenience, and snowball sampling approaches, starting with the individuals most familiar with the intervention and the effectiveness trial, including intervention recipients (i.e., trial participants, such as patients in a clinic, community members, etc.) and intervention deliverers (e.g., providers in a clinic, lay health workers in the community, research staff). These participants can help identify other individuals and groups for researchers to recruit (i.e., snowball sampling). As with the first goal, the sampling for the second goal typically uses principles of maximum variation and saturation.

Sampling can be augmented and extended by using theoretical sampling approaches and determinant frameworks such as CFIR, which can guide expanding the sampling frame to several other groups that were less directly involved with the intervention, but who may be able to provide key information for future implementation. First, we can consider including individuals from the broader socio-ecological spheres of both the recipients (e.g., their families and other social supports) and the providers (e.g., their teams, leadership), as relevant for a given intervention and setting. Sampling can extend beyond the organizational inner context into the outer context (environment), including key payers (e.g., insurance companies, government agencies), purveyors and intermediaries (e.g., pharmaceutical companies, technical assistance providers), regulatory and policy actors (e.g., licensing boards, government officials), and other individuals and organizations at the community, state, or federal levels. Second, we can consider expanding the samples to include places and individuals that were not included in the trial but represent settings and communities where future implementation efforts are likely to occur. While these individuals will not have direct experience with the intervention (we thus refer to them as “naïve” participants), they do have relevant knowledge of their context and can provide data on *anticipated* determinants in their contexts/settings that can be used to inform future implementation efforts.

Qualitative data are typically collected using individual interviews, which allow for collecting rich data on individual perceptions and are relatively feasible to schedule, one participant at a time (particularly for clinicians and other professionals)). However, in some cases, focus groups (or group interviews) are a viable alternative, particularly when the eligible group is numerous (e.g., patients in a community, nurses in a hospital), or individuals already gather for other purposes (e.g., teachers meeting, a community event for individuals with a relevant diagnosis). Both individual interview and focus group questions are typically structured around the chosen frameworks to ensure the conversations systematically and rigorously cover a broad range of topics (e.g., perceptions of the intervention, the context, health equity). Quantitative data collection (i.e., surveys) can augment qualitative data collection in the same sample (e.g., qualitative interviews+brief surveys of acceptability, feasibility, and appropriateness), or it can extend the sample where a larger sample of individuals is surveyed (e.g., all teachers in all schools where intervention was tested) and a subsample is interviewed (e.g., five teachers per school) for more in-depth, rich exploration of their experiences.

#### Other considerations

A study of implementation determinants is relatively straightforward and can be achieved with limited resources. With guidance and consultation of an implementation scientist, design, data collection, and analyses may often be conducted by other members of the research team (e.g., trained research staff or postdocs) with foundational knowledge of implementation science theories and methods. In some cases, a senior member of the research team (e.g., study lead) with an established reputation or ties to the participants and their communities may need to help obtain high-level approvals (e.g., with clinic or school leadership) before individuals can be recruited, and in some instances a more senior, experienced researchers may be best suited to conduct certain interviews of focus groups (e.g., with executive leadership, physicians), depending on the local norms and hierarchy.

### Goal 3: examine stakeholder perceptions to inform the effectiveness trial

#### Purpose and research questions

Finally, the third goal flips the chronological order of the aims, placing the implementation aims before the intervention effectiveness trial to inform intervention development, adaptation, or bundling (i.e., combining different interventions/components), development of strategies to support conducting the effectiveness trial (e.g., training, reminders, technical assistance/coaching), or research/evaluation methods (e.g., effective recruitment of participants in a marginalized community, selection of outcomes most relevant to key partners). Such hybrid studies begin with answering questions around both intervention determinants (e.g., What barriers to accessing/using the intervention do recipients anticipate experiencing? What intervention design would be most appealing?) and implementation determinants (e.g., How to design the intervention for feasibility and sustainability in real-world settings? How to design training for providers?) before an effectiveness trial. This approach is also ideally suited to include health equity considerations at the intervention design stage. We recognize that other researchers have proposed similar approaches to intervention development (51–53). Our intent is to highlight these considerations and link them to our current thinking on hybrid study approaches.

We also recognize that in many cases, this approach would likely require a pilot study to precede the fully-powered intervention effectiveness study, which can be designed as a standalone hybrid type 1 *pilot* study [e.g., NIH R21 (54) or R34 (55) mechanism, or even R01 (34)] or as a series of hybrid studies using bi-phasic funding approaches [e.g., NIH UG3/UH3 (54, 56), or R61/R33 (57, 58)]. Piloting is particularly critical in studies involving extensive intervention development or adaptation, prototyping, and iteration. For hybrid type 1 pilot studies, it would be appropriate for the implementation-focused work to center more on the characteristics of the intervention, and less on the implementation context. If the study team follows up the pilot study with a fully-powered type 1 intervention effectiveness study, it would make sense to for the follow-up, larger study to focus more explicitly and fully on the implementation context (inner and outer) using one or both of the other two hybrid type 1 goals. Finally, in some cases it may be feasible to use the goal 3 approach before an intervention effectiveness study without a pilot phase, particularly in studies bundling well-researched interventions/components, when adaptations are relatively minor (adaptable periphery), or in studies relying on more pragmatic and adaptable designs, such as quality improvement studies.

It is important to recognize that goal 3 is a relatively new idea that will require additional refinement through application. However, we and other researchers have used elements of this approach in several proposal and studies, often not even under the banner of hybrid approaches. For an example, see the protocol paper by Decker et al. (59) that is also highlighted in the Supplementary File S2 that presents exemplars of hybrid approaches.

#### Evaluation design and framework

As outlined in the previous paragraph, in this design the implementation aim precedes the intervention aim, significantly increasing the level of interaction between implementation and effectiveness aims. Of note, in the case of a hybrid type 1 *pilot* study (or phase) preceding a larger trial, the two aims of the hybrid pilot could follow any number of sequences, including the implementation → effectiveness sequence (I→E), a more typical E→I sequence, or the two aims could be highly interwoven and executed in a series of iterative cycles such as I→E→I→E. However, we would argue that this approach does not increase the risks to the intervention effectiveness aim (trial integrity, contamination, etc.), but can indeed minimize associated risks by ensuring the intervention, strategies, and other methods are well-designed and have a high potential for future implementability and scalability in real-world settings, thus maximizing the long-term impact of the intervention.

To achieve the aims associated with this approach, beginning the study with a formative evaluation facilitates obtaining the most relevant input from key groups, including intervention recipients (e.g., patients in health settings, students or families in schools) and intervention deliverers (professionals who would deliver the intervention in the trial and later in routine practice, such as providers in clinics, or teachers in schools). To that end, several frameworks mentioned earlier [e.g., determinant frameworks such as CFIR (5, 60)] would be helpful. They could be extended with individual-focused theories, models, and frameworks [e.g., Theoretical Domains Framework (61, 62), Health Belief Model (63, 64), Andersen's Behavioral Model (65)] and frameworks focused on health disparities and health equity [e.g., NIMHD Research Framework (66), health equity implementation framework (46)] to broaden the considerations for intervention design and accessibility in real-word settings. Finally, several process models and frameworks for intervention *adaptation* [e.g., ADAPT-ITT (67), FRAME (68)] and *development/design* (Design for Dissemination and Sustainment/D4DS (51), PRECEDE-PROCEED model (69), intervention mapping (70), user/human-centered design (52, 53)) can be used to guide formative evaluation and integration of the findings in the effectiveness aim.

#### Sampling and data collection

Similar to the other two goals above, the primary sampling frame for the third goal includes *anticipated* intervention recipients (e.g., patients, students) and deliverers (e.g., providers, teachers) who have the most relevant knowledge of their contexts, needs, and capacities, providing the necessary information that can inform the design of the intervention and strategies for implementing it.

Furthermore, and similar to the second goal, researchers could consider including other relevant individuals and groups, such as purveyors, technical assistance providers, payers, or policy makers. Considering that these groups are essential for supporting the intervention implementation and scale up in the future, gathering their perceptions and input early on could provide helpful information regarding intervention design (e.g., cost, complexity, feasibility). For example, potential technical assistance providers could provide input on intervention complexity (based on their ability to support it), or payers could provide input on cost targets (based on their willingness to pay). While adding such considerations may be informative and reduce the need for future adaptation, we also recognize that in some cases it may be premature or unfeasible, particularly at the pilot phase.

Finally, data collection follows a similar approach as goal two, with a bigger emphasis on qualitative methods to capture individuals' determinants, preferences, and recommendations for intervention design. Should researchers adopt a user-centered design approach to (re)designing the intervention, additional behavioral and/or quantitative data could be obtained from usability-testing and prototyping various components before conducting the intervention effectiveness trial. Importantly, the researchers should also consider what factors related to health equity need to be examined to ensure that the needs of diverse groups are met.

#### Other considerations

When considering using the third goal for designing and conducting an effectiveness study, including adding a pilot phase or a standalone pilot study, the research team needs to consider available resources and team structure and expertise. While this approach offers many potential benefits, it can significantly lengthen the timeline, often necessitate pilot studies (or phases), increase resource use, and require a larger, well-coordinated team of intervention and implementation experts. That being said, the implementation aims can vary in scope and intensity, and while some approaches may require significant intervention (re)design, prototyping, and piloting of components, some studies could proceed relatively quickly to the effectiveness aim. For example, formative data could be used to inform changes to the intervention's adaptable periphery, design a menu of options for intervention deliverers [i.e., forms and functions approach to intervention/trial design (71, 72)], or to inform optimization or adaptive trial designs [e.g., Sequential Multiple Assignment Randomized Trial or SMART, Multiphase Optimization Strategy or MOST (73)].

### Tools and resources to help you design a hybrid type 1 study

#### Hybrid type 1 study planning tool

To assist researchers in planning hybrid type 1 studies, we developed a “Hybrid Type 1 Effectiveness-Implementation Study Planning Tool” (see Supplementary File S1) adapted from other resources/guidance for planning and evaluating research studies with enhanced attention to implementation factors (41, 74, 75). The planning tool offers pertinent considerations and recommendations for each of the three hybrid type 1 goals (as described above) and is arranged accordingly in tabular format with one column dedicated to each of the three goals.

Consistent with a recent scoping review (28) indicating that the RE-AIM evaluation framework (41) has been the most commonly used in hybrid type 1 studies, the planning tool organizes considerations/recommendations for outcomes assessment by RE-AIM dimensions (i.e., reach, effectiveness, adoption, implementation, maintenance). Though RE-AIM was used as an organizing framework for outcomes assessment in the planning tool given its pervasive use within hybrid type 1 studies, researchers obviously may choose to use an alternative evaluation framework for their hybrid type 1 study if they prefer.

Because the planning tool is arranged according to hybrid type 1 goal, the considerations/recommendations for one goal in relation to the others may appear to overlap or be redundant. This is due to the temporal quality of the effectiveness- and implementation-focused questions addressed within each goal (e.g., in/during vs. after vs. before trial), which introduces subtle but important distinctions that have implications for hybrid type 1 study questions and planning. To the extent possible, we have attempted to minimize overlap/redundancy by providing greater detail on considerations/recommendations in one column of the planning tool and referring to that additional detail in the other columns (without duplicating it). Further, because some hybrid type 1 studies focus on multiple goals in combination (particularly goals 1 and 2), researchers may need to refer to multiple columns in the tool in planning their study.

Finally, while the hybrid type 1 planning tool is comprehensive, it is not rigidly prescriptive. Investigators are encouraged to review the considerations and recommendations provided in the tool and integrate those they feel best fit the research question(s) for their hybrid type 1 study and which are logistically feasible to address within their study's scope and available resources.

#### Annotated bibliography of hybrid type 1 study exemplars

In Supplementary File S2, we provide an “Annotated Bibliography of Hybrid Type 1 Effectiveness-Implementation Study Exemplars” which lists and summarizes protocol papers for five select exemplar hybrid type 1 studies. For each of the five hybrid type 1 exemplar studies cited, the bibliography provides summary information (annotations) to describe the effectiveness- and implementation-focused methods from the protocol paper, along with references to specific page numbers within the cited articles where additional methodological detail can be found. Because the resource is intended for use by effectiveness researchers interested in learning more about planning for and applying implementation-focused methods in hybrid type 1 studies, the annotations purposefully give greater attention to summarizing the *implementation-focused* methods in the study exemplars.

#### Additional resources

- A rich collection of tools and resources for applying the RE-AIM framework (41) is accessible at http://www.re-aim.org, including guidance and examples for how to apply them.
- Given that our hybrid type 1 planning tool recommends use of a determinant framework (25) for conceptualizing and assessing implementation barriers and facilitators in hybrid type 1 studies, we highlight resources available for the two most commonly used in hybrid type 1 studies (28) - the “Consolidated Framework for Implementation Research” (CFIR) (5, 76) and the integrated—Promoting Action on Research Implementation in Health Services (i-PARIHS) (6) framework. CFIR developers offer an online “guide” (https://cfirguide.org/) where researchers can find guidance, tools, and other resources for applying the framework. For those interested in using i-PARIHS, Ritchie et al. (77) developed a publicly-available codebook to guide qualitative data analyses for assessing implementation barriers and facilitators according to i-PARIHS constructs (downloadable as “Additional File 2” at https://pmc.ncbi.nlm.nih.gov/articles/PMC9476709/).
- In the “Applicant and Awardee Resources” section of their website (78), the Patient-Centered Outcomes Research Institute (PCORI) describes their expectations for aims/outcomes and additional methodological guidance for all hybrid study types (https://www.pcori.org/funding-opportunities/applicant-and-awardee-resources/frequently-asked-questions/hybrid-studies).
- Finally, for those who may have a broader interest in implementation science methods, a 2020 *Psychiatry Research* special issue on implementation science (https://www.sciencedirect.com/journal/psychiatry-research/vol/283/suppl/C) included a number of papers with information that may be relevant to enhancing implementation assessment in hybrid type 1 studies, including an introduction to hybrid studies (1) quantitative (38) and qualitative (37, 39) methods in implementation research (IR), use of theory in IR (40), and an introduction to implementation strategies (79).

## Discussion

In this paper, we provide an overview of effectiveness-implementation hybrid type 1 studies, identify three goals of implementation aims, and provide a planning tool and other resources that can support and augment the design of effectiveness studies to maximize the (future) impact of interventions. We recommend that effectiveness researchers consider incorporating one or more of these goals into their studies, depending on the state of evidence for their intervention, research questions that need to be answered, and the resources available (funding, staff, expertise). We argue that goals 1 and 2, which are relatively common, low in resource intensity, and pose minimal risk, are appropriate and feasible in most cases. The two goals are also compatible, so the team could choose to pursue both at the same time, or chose one or the other depending on resources available, risk, and timing. The third goal is somewhat newer (less common) and more resource-intensive, but offers an implementation-focused approach to intervention development, adaptation, or bundling, and may be particularly well-suited for pilot studies. While the three goals present a menu of options for researchers to consider, we stress that the goals are (1) depicted more as “ideal types” but are flexible in operationalization, (2) not mutually exclusive, but rather compatible and adaptable to fit the study, and (3) that new goals and combinations of goals could be identified in the future.

This article extends the guidance and considerations on designing hybrid type 1 studies currently provided in the literature (13, 17, 80). Furthermore—and depending on researchers on the team, their discipline and expertise, and their interests—hybrid type 1 studies could be designed for use with other methods in subsequent steps. For example, the approach our teams typically take is to use hybrid type 1 findings to inform a partner-engaged process [EBQI (81)] where we typically discuss the findings (e.g., determinants, recommendations), identify and prioritize most salient determinants, select and tailor implementation strategies to address them, and (if applicable) adapt the intervention, all of which then informs the next implementation study. Such partner-engaged approaches can be augmented with group methods such as Delphi, Nominal Group Technique, Concept Mapping, or Group Model Building. When resources (e.g., time, funding) allow, conducting such activities may be conducted as part of the same hybrid type 1 study (7), or in the beginning phases of the next study. Follow-up aims or studies could also investigate hybrid type 1 findings in a larger, survey-based study; for example, researchers could investigate how common the identified implementation determinants are in a nationally representative sample. This is particularly appropriate for relatively established interventions, such as medication-assisted treatment for substance use disorders or the diabetes prevention program, that are familiar to practitioners but underutilized. Finally, hybrid type 1 studies can also provide data for conducting economic evaluations (e.g., cost effectiveness, budget impact analysis), which can either guide further research (e.g., adapt intervention for lower cost) or directly inform leaders to adopt the EBI in routine practice.

Hybrid study methods align favorably with Learning Health System (LHS) (82) principles in the clinical domain, and with community-based participatory research (83) (CBPR) approaches in the community and public health domains. Both encourage use of participatory approaches (83, 84) to engage end-users and other key stakeholders (e.g., intervention deliverers) to systematically elicit their perspectives/preferences and integrating that data into intervention design/adaptation, with the ultimate goal of increasing the intervention's real-world relevance, feasibility, and impact in routine practice. For example, the U.S. Veterans Health Administration promotes use of LHS and engagement science approaches to more systematically involve patients, providers, healthcare leaders, and other interested parties in all stages of its intramural research program in efforts to ensure that effective treatments, programs, and policies are more rapidly developed and implemented in routine practice to maximize real-world impact for end-users (85, 86). Similarly, CBPR approaches emphasize the involvement of a wide range of community partners in the design and conduct of effectiveness and implementation research to enhance relevance, rigor, and impact of the research (87). Hybrid type 1 studies fit these research traditions, and we think that this paper can help inform and advance such studies to help increase the likelihood of achieving the desired population health impact. At the same time, hybrid type 1 researchers could learn from these approaches to enhance the design and conduct of their research in novel ways, particularly as these approaches continue to develop.

Hybrid type 1 studies are also well-positioned to move the field beyond simply documenting implementation determinants and advance our understanding of implementation processes by incorporating more theory use and theorizing (88, 89), and investigating intervention and implementation mechanisms (90), which is increasingly the expectation for publishing studies on implementation determinants (91). The process-oriented analyses (goals 1 and 2) are well-suited for that purpose. Such efforts could include deductive approaches (e.g., testing theories), inductive/grounded theory approaches (e.g., developing theories), or abductive approaches (hypothesizing what theories explain observed patterns). Hybrid type 1 studies could also help explore and map mechanisms using methods such as causal pathway diagrams (92), causal loop diagrams (93), or directed acyclic graphs (94). These efforts to map the mechanisms could then inform the design and evaluation of follow-up studies.

In summary, we hope this paper will inform future effectiveness research and expand the use of hybrid type 1 approaches, which can accelerate adoption, scale, and population health impact of evidence-based interventions by investigating “implementability” earlier in the intervention development pipeline. While the use of hybrid type 1 approaches has increased in recent years, opportunities to expand and enhance their use remain. We identified and articulated three implementation-focused goals in hybrid type 1 studies, which offer a menu of options for effectiveness researchers to integrate into their studies. At the same time, we hope this paper also informs implementation scientists and current users of hybrid type 1 approaches to consider a broader range of questions and methods than they currently employ in their work. Finally, we would like to reiterate that new goals and methods for implementation aims in hybrid type 1 studies may be articulated or developed in the future. Such innovations are welcome and needed. Together, these advances have the potential to improve intervention design and increase their uptake, sustainment, scale, and impact on population health.

## Data Availability

The original contributions presented in the study are included in the article/Supplementary Material, further inquiries can be directed to the corresponding author.
