# Supplementary material for: Hybrid type 1 effectiveness-implementation studies: why and how to do them
Source: Front Health Serv. 2026 Mar 5;6:1678257. doi: 10.3389/frhs.2026.1678257 (PMC12999578; doi:10.3389/frhs.2026.1678257)
Supplement: Supplementary file 1 [file Supplementaryfile1.pdf]

Supplemental File 1 Hybrid Type 1 Effectiveness-Implementation Study Planning Tool

Overview

- The purpose of the 'Hybrid Type 1 Effectiveness-Implementation Study Planning Tool' (next page) is to assist researchers in planning and conducting hybrid type 1 studies. The planning tool offers pertinent considerations and recommendations for each of the three hybrid type 1 goals (see below) and is arranged accordingly in tabular format with one column dedicated to each of the three goals.

  <u>Hybrid Type 1 Goals</u>
  1) Explain intervention implementation in the effectiveness trial
  2) Explore stakeholder perceptions to inform future implementation research
  3) Examine stakeholder perceptions to inform the effectiveness trial

  - **<u>IMPORTANT!</u>**
    While this hybrid type 1 planning tool is comprehensive, it is not rigidly prescriptive. Researchers are encouraged to review the considerations and recommendations provided in the tool and integrate those they feel best fit the research question(s) for their hybrid type 1 study and which are logistically feasible to address within their study's scope and available resources.

  - **<u>NOTES:</u>**
    - Because the planning tool is arranged according to the three hybrid type 1 goals, the considerations/recommendations for one goal in relation to the others may in some cases appear to overlap or be redundant. This is due to the temporal quality of the effectiveness- and implementation-focused questions addressed within each goal (*e.g., in/during vs. after vs. before effectiveness trial*), which introduces subtle but important distinctions that have implications for study planning. To the extent possible, we have attempted to minimize overlap/redundancy by providing greater detail on considerations/recommendations in one column of the planning tool and referring to that additional detail in the other columns (without duplicating it).

    - Finally, because some studies may focus on multiple hybrid type 1 goals in combination, researchers may need to refer to multiple columns in the tool in planning their study.

  - This tool was informed by and/or adapted from:

    - Glasgow RE, Harden SM, Gaglio B, et al. RE-AIM planning and evaluation framework: Adapting to new science and practice with a 20-year review. *Front Public Health* 2019; 7:64. www.re-aim.org
    - Chasco EE, Van Tiem J, Johnson N, et al. RE-AIM for rural health innovations: perceptions of (mis) alignment between the RE-AIM framework and evaluation reporting in the Department of Veterans Affairs Enterprise-Wide Initiatives program. *Front Health Serv* 2024; 4:1278209.
    - Kowalski CP, Kawentel LM, Kyriakides TC, et al. Facilitating future implementation and translation to clinical practice: The Implementation Planning Assessment Tool for clinical trials. *J Clin Transl Sci* 2022; 6(1):e131.

Goal	1) Explain intervention implementation in the effectiveness trial	2) Explore stakeholder perceptions to inform future implementation research	3) Examine stakeholder perceptions to inform the effectiveness trial
**Intervention**	*Describe the intervention as being implemented; include information on the setting(s) where the intervention is being delivered and the types of clinical and/or non-clinical staff (by discipline, position, etc.) involved in delivering it.*<br>• See the 'Implementation' row below regarding assessment of fidelity to core functions and forms of the intervention.<br>• See also the 'Reach', 'Effectiveness', 'Adoption', and 'Maintenance' rows below regarding the need to document any modifications/adaptations made to the intervention to try to enhance impact on those domains.	*Describe the intervention as implemented; include information on the setting(s) where the intervention was delivered and the types of clinical and/or non-clinical staff (by discipline, position, etc.) who were involved in delivering it.*<br>• See the 'Implementation' row below regarding assessment of fidelity to core functions and forms of the intervention.<br>• See also the 'Reach', 'Effectiveness', 'Adoption', and 'Maintenance' rows below regarding the need to document any modifications/adaptations made to the intervention to try to enhance impact on those domains.	*Describe the developed/adapted intervention to be tested (e.g., in a hybrid type 1 pilot study). Include information on the setting(s) where the intervention will be delivered and the types of clinical and/or non-clinical staff (by discipline, position, etc.) who will be involved in delivering it.*<br>• As feasible in developing/adapting the intervention, consider identifying core functions and forms for implementing the intervention with fidelity.
**Framework, model, theory (FMT)**	*Specify the framework, model, and/or theory guiding implementation of the intervention and evaluation of its use/impact. Include an explanation of the rationale for the FMT(s) selected.*<br>• Consider complementary use of a determinant framework (e.g., CFIR, i-PARIHS) and evaluation framework (e.g., RE-AIM) to guide implementation assessment and evaluation<br>• Determinant frameworks propose domains and constructs that can be used to guide and inform assessment of barriers and facilitators to intervention implementation (see the 'Implementation' row below). Consider exploring determinants pertaining to context, characteristics of intervention deliverers and recipients, and characteristics of the innovation itself. CFIR and i-PARIHS are frequently used to assess such factors in hybrid type 1 studies. In selecting a framework, consider also whether one which gives greater attention to health equity domains/constructs (eg, PRISM, Health	*Specify the framework, model, and/or theory that guided implementation of the intervention and evaluation of its use/impact. Include an explanation of the rationale for the FMT(s) selected.*<br>• See 2nd column for additional information and considerations.	*Specify the framework, model, and/or theory that will guide intervention development/adaptation and pilot-testing. Include an explanation of the rationale for the FMT(s) selected.*<br>• See 2nd column for additional information and considerations.

Goal	1) Explain intervention implementation in the effectiveness trial	2) Explore stakeholder perceptions to inform future implementation research	3) Examine stakeholder perceptions to inform the effectiveness trial
	Equity Implementation Framework (HEIF)) may better address study questions or guide analyses.<br>• Dimensions and guidance within an evaluation framework can be used to inform selection of study outcome measures. RE-AIM is a frequently used evaluation framework in hybrid type 1 studies (see 'Outcomes' section below).		
**Outcomes***			
Reach	*Assess the absolute number, proportion, and representativeness of those receiving the intervention.*<br>• Is the intervention being delivered to the target population as planned?<br>• Are specific subgroups of the targeted population more likely to receive the intervention than others? Which and why? Such questions relating to health equity allow examination of whether receipt of the intervention is equitable and does not result in care disparities (e.g., as an unintended consequence).<br>• What strategies (if any) are being used to overcome challenges and increase reach to the targeted population?<br>• Is the intervention being delivered to individuals *not* included in the planned target population? If yes, what implications (if any) is this having on reach to the targeted population (e.g., value added?, opportunity costs?)?	*Assess the absolute number, proportion, and representativeness of those who received the intervention.*<br>• Was the intervention delivered to the target population as planned?<br>• Were specific subgroups of the targeted population more likely to receive the intervention than others? Which and why? Such questions relating to health equity allow examination of whether receipt of the intervention is equitable and does not result in care disparities (e.g., as an unintended consequence).<br>• What strategies (if any) were used to overcome challenges and increase reach to the targeted population?<br>• Was the intervention delivered to individuals *not* included in the planned target population? If yes, what implications (if any) did this have on reach to the targeted population (e.g., value added?, opportunity costs?)?	*Specify plans for assessing the absolute number, proportion, and representativeness of individuals who are intended to receive the intervention.*<br>• In piloting the intervention, who is intended to benefit (e.g., patients, clients, community members), and who will actually participate or be exposed to the intervention (i.e., target population to receive the intervention)?
Effectiveness	*Assess the intervention's impact on primary and secondary measures of effectiveness (e.g., improvement in processes of care, patient symptoms, functioning / quality of life, disease prevention, economic outcomes (e.g., cost-effectiveness)).*<br>• Are anticipated (hypothesized) outcome improvements being achieved?	*Assess the intervention's impact on primary and secondary measures of effectiveness (e.g., improvement in processes of care, patient symptoms, functioning / quality of life, disease prevention, economic outcomes (e.g., cost-effectiveness)).*<br>• Were anticipated (hypothesized) outcome improvements achieved?	*Specify primary and secondary outcome measures to assess effectiveness of the developed/adapted intervention; data sources or data collection methods to be used; and planned data analysis methods.*<br>• In piloting the intervention, what are the benefits you will be trying to achieve, as measured by change on key measures of

Goal	1) Explain intervention implementation in the effectiveness trial	2) Explore stakeholder perceptions to inform future implementation research	3) Examine stakeholder perceptions to inform the effectiveness trial
	• Are specific subgroup populations or settings differentially benefiting from the intervention (e.g., in terms of improved care/outcomes)? Which and why? Such questions relating to health equity allow examination of whether benefits from the intervention are equitable and do not result in outcomes disparities (e.g., as an unintended consequence).<br>• Have any negative outcomes emerged? If yes, how are these being addressed or alleviated?<br>• What modifications /adaptations (if any) are being made to the intervention to either enhance its effectiveness or eliminate/reduce any negative outcomes?	• Did any specific subgroups differentially benefit from the intervention (e.g., in terms of improved care/outcomes)? Which and why? Such questions relating to health equity allow examination of whether benefits from the intervention are equitable and do not result in outcomes disparities (e.g., as an unintended consequence).<br>• Were any negative outcomes observed? If yes, how were those addressed/alleviated; or how might they be addressed/alleviated in future implementation efforts?<br>• What modifications /adaptations (if any) were made to the intervention during the trial to either enhance its effectiveness or eliminate/reduce any negative outcomes?	effectiveness (e.g., improvement in processes of care, patient symptoms, functioning / quality of life, disease prevention) or economic outcomes (e.g., cost-effectiveness)?<br>• In piloting the intervention, how will you assess issues pertaining to health equity (e.g., whether specific subgroups may differentially benefit in terms of receipt of or outcomes from the intervention)?<br>• Also, what is the likelihood for potential negative outcomes and how might these be avoided or alleviated?
Adoption	*Adoption is measured in terms of which eligible settings or staff take up the intervention and deliver it, and which do not. Assess the absolute number, proportion, and representativeness of settings or staff delivering the intervention. Specify how this is being measured.*<br>• How many and what proportion of eligible sites or providers, frontline staff, etc., are delivering the intervention?<br>• How do the sites/providers/etc. delivering the intervention differ from others who are eligible but are either not delivering it or not delivering it to the same extent?<br>• What challenges or obstacles are preventing sites/providers/etc. from delivering the intervention?<br>• What strategies (if any) are being used to overcome challenges and increase adoption among participating sites/providers/etc.?	*Assess the absolute number, proportion, and representativeness of settings or staff that delivered the intervention. Specify how this was measured.*<br>• How many and what proportion of eligible sites or providers, frontline staff, etc., delivered the intervention?<br>• How do the sites/providers/etc. that delivered the intervention differ from others who were eligible but either did not deliver it or did not deliver it to the same extent?<br>• What challenges or obstacles prevented sites/providers/etc. from delivering the intervention?<br>• What strategies (if any) were used to overcome challenges and increase adoption among participating sites/providers/etc.?<br>• What implications do findings pertaining to adoption have for future implementation of the intervention?	*In piloting the intervention, specify plans for assessing the absolute number, proportion, and representativeness of settings and staff intended to deliver the intervention. Include information on data sources or data collection methods to be used, and planned data analysis methods.*<br>• See columns to the left for additional information and considerations about assessing intervention adoption.

Goal	1) Explain intervention implementation in the effectiveness trial	2) Explore stakeholder perceptions to inform future implementation research	3) Examine stakeholder perceptions to inform the effectiveness trial
Implementation	• Is the intervention being delivered with fidelity to its core functions and forms and any site-specific implementation goals? How is this being assessed (e.g., data sources, EHR documentation)?<br>• What adaptations, if any, have been made to the intervention and/or strategies/tools used in implementing it?<br>• Are effectiveness measures (see above) being impacted by differences in intervention fidelity and/or adaptations; and to what extent?<br>• Are there differences across sites in implementing the intervention? If so, how and why are they differing?<br>• What barriers are being encountered in implementing the intervention?<br>○ Which barriers are being commonly encountered across sites? Which barriers are more site- or context-specific?<br>○ What strategies, if any, are being employed to try to overcome these barriers?<br>• What facilitators are being encountered that are helping to enable implementation of the intervention?<br>• What resources (including cost) are associated with implementing the intervention, and how are they being assessed and documented?	• Was the intervention delivered with fidelity to its core functions and forms and any site-specific implementation goals? How was this assessed (e.g., data sources, EHR documentation)?<br>• What adaptations, if any, were made to the intervention and/or strategies/tools used in implementing it?<br>• Were effectiveness measures (see above) impacted by differences in intervention fidelity and/or adaptations, and to what extent?<br>• Did sites differ in implementation of the intervention? If so, how and why did they differ?<br>• What barriers were encountered in implementing the intervention?<br>○ Which barriers were commonly encountered across sites? Which barriers were more site- or context-specific?<br>○ What strategies, if any, were employed to try to overcome these barriers?<br>• What facilitators were encountered that helped enable implementation of the intervention?<br>• What resources (including cost) were associated with implementing the intervention, and how were they assessed and documented?<br>• Based on project findings, what specific implementation strategies/tools will be required to successfully implement the intervention in future efforts?	• In piloting the intervention, describe plans for ensuring the intervention will be implemented with fidelity to its core functions and forms; and how this will be assessed (specify data sources, EHR documentation requirements, etc.).<br>• Describe what types of adaptations, if any, may be made to the intervention and/or strategies/tools used in implementing it.<br>• Describe how you will assess whether there are any differences across sites in implementing the intervention?<br>• Describe how you will assess whether effectiveness measures (see above) are impacted by differences in intervention fidelity and/or adaptations.<br>• Describe plans for assessing barriers (challenges) and facilitators (enablers) to implementing the intervention; and if/how the intervention or implementation approach may be adapted during the pilot study to address barriers and/or leverage facilitators.<br>• Describe any plans for assessing what resources (including cost) are required to implement the intervention with fidelity.
Maintenance (sustainment)	*Maintenance refers to the extent to which the intervention is sustained or 'institutionalized', where its continued use becomes part of routine organizational practices and policies.*	• At the conclusion of the study, assess whether and to what extent use and impact of the intervention was sustained.<br>○ Repeat measures of Reach, Effectiveness, and/or Adoption used during the study to assess whether	• In piloting the intervention, describe any plans to assess whether and to what extent use and impact of the intervention will be sustained in later stages of the study or after the study ends.

Goal	1) Explain intervention implementation in the effectiveness trial	2) Explore stakeholder perceptions to inform future implementation research	3) Examine stakeholder perceptions to inform the effectiveness trial
	• In later stages of the study, assess whether and to what extent use and impact of the intervention is being sustained.<br>o Repeat measures of Reach, Effectiveness, and/or Adoption used in earlier stages of the study to assess whether innovation use and impact is maintained or enhanced in later stages; specify when and how these measures are assessed.<br>o Assess sustained use and impact of the intervention at ‘setting’, ‘provider/staff’, and/or ‘patient/client’ levels, as applicable.<br>• If use/impact of the intervention is not being sustained, ask site leaders/providers/staff (in surveys, qualitative interviews, or focus group(s)) what resources, tools, or support may help improve their sustained use of the intervention.	innovation use and impact was maintained or enhanced; specify when and how these measures were assessed.<br>o Assess sustained use and impact of the intervention at ‘setting’, ‘provider/staff’, and ‘patient/client’ levels, as applicable.<br>• Ask site leaders/providers/staff (in qualitative interviews, surveys, focus group(s)) whether they plan to continue use of the intervention in routine practice after the study ends; why or why not?<br>o If yes, ask what preparation or planning has been done at the site(s) to support continued use of the intervention?<br>o If no, ask site leaders/providers/staff what additional resources, tools, policies, or support might help them sustain use of the intervention now that the study is ending.<br>• Has the site (or sites) developed/identified measures/metrics that they will use to assess use and impact of the intervention on an ongoing basis once the study ends? Or, for the research team, can data infrastructure and analytic methods used by the study team be ‘handed off’ to sites for their continued use in monitoring delivery/impact of the intervention (e.g., dashboards)?	o If measures of Reach, Effectiveness, and/or Adoption will be repeated in later stages of the study or after the study ends for assessing the intervention’s sustained use and impact, describe when and how these will be assessed.<br>o In planning, consider including measures to assess sustained use and impact of the intervention at ‘setting’, ‘provider/staff’, and ‘patient/client’ levels, as applicable.<br>• At minimum, consider planning to ask site leaders/providers/staff (in qualitative interviews, surveys, or focus group(s) at the end of the pilot study) whether they plan to continue use of the intervention in routine practice; and why or why not?

* Outcomes dimensions (reach, effectiveness, adoption, implementation, maintenance) are drawn from:

- Glasgow RE, et al. RE-AIM planning and evaluation framework: Adapting to new science and practice with a 20-year review. *Front Public Health* 2019; 7:64.
