# Supplementary material for: Hybrid type 1 effectiveness-implementation studies: why and how to do them
Source: Front Health Serv. 2026 Mar 5;6:1678257. doi: 10.3389/frhs.2026.1678257 (PMC12999578; doi:10.3389/frhs.2026.1678257)
Supplement: Supplementary file 2 [file Supplementaryfile2.pdf]

Supplemental File 2
Annotated Bibliography
of Hybrid Type 1 Effectiveness-Implementation Study Exemplars

This bibliography lists protocol papers for five select exemplar effectiveness-implementation hybrid type 1 studies. Each citation is annotated to provide brief summary information on the effectiveness- and implementation-focused methodology within each study, with references to specific page numbers within each article where additional methodological detail can be found. The bibliography includes two exemplars conducted in Department of Veterans Affairs (VA) healthcare settings and three exemplars conducted in non-VA healthcare or community settings. Because this resource is intended for use by established efficacy/effectiveness researchers interested in learning more about planning for and applying implementation-focused methods in effectiveness-implementation hybrid type 1 studies, the annotations purposefully give greater attention to summarizing the implementation-focused methods in these studies.

VA Exemplars

1. **Decker SE, Kroll-Desrosiers A, Mattocks KM, et al. Study protocol of a hybrid type 1 effectiveness-implementation multisite trial: Dialectical behavior therapy skills group for Veterans at high-risk for suicide attempt. *Contemporary Clinical Trials* 2025; 151:107828. https://doi.org/10.1016/j.cct.2025.107828**

    - **Hybrid Type 1 Goals Addressed:** Goals 1, 2, and 3

    - **Objective:** Dialectical Behavior Therapy (DBT) is effective for reducing suicide attempts in individuals with emotional dysregulation and repeat suicidal behaviors or self-directed violence but is resource intensive. A more resource-efficient component of DBT, the DBT Skills Group (DBT-SG) has been shown to be as efficacious as comprehensive DBT in non-veteran samples, but its effectiveness and factors affecting its implementation have not been studied in the Veterans Health Administration (VHA). Study aims are to: test DBT-SG's effectiveness for reducing emotion dysregulation among high-risk Veterans with previous suicide attempts; and identify implementation barriers and facilitators of DBT-SG in VHA.

    - **Design:** Multi-site randomized controlled trial with a concurrent formative evaluation of barriers and facilitators to DBT-SG implementation at each study site.

    - **Setting:** Four VHA medical centers

    - **Intervention:** DBT-SG is a structured group skills training intervention teaching skills in four modules following a written manual. Two-hour group sessions are led by group leaders who balance acceptance and change approaches. Group is taught like a class using a psychoeducational format, and session agenda includes mindfulness practice, review of prior session's homework, and new skills instruction. Participants are encouraged to seek additional mental health support for other needs not typically addressed in group, such as medication management, trauma processing, or case management. Group is supported by a weekly one-hour therapist consultation team meeting following a structured agenda.

- **Summary of Hybrid Type 1 Study Methodology**
  Effectiveness: Primary effectiveness outcome is emotion dysregulation, which is associated with veteran suicide attempt and hospitalization. Secondary effectiveness outcomes include number of suicide attempts (including aborted or interrupted attempts), time to first suicide attempt, suicidal ideation, and coping skills. See sections 2.4.7 – 2.4.9 on pp. 5-6 of the article for additional information on effectiveness assessments and analysis plans.

  Implementation: To identify DBT-SG implementation barriers and facilitators, formative evaluation guided by the *integrated – Promoting Action on Research Implementation in Health Services (i-PARIHS)* framework will involve developmental (pre-trial), process (during trial), and post-trial data collection. At each of the three formative evaluation intervals, the study team will conduct qualitative interviews until thematic saturation is reached (upper limit of 60 total interviews) with both VA staff and veterans. For the developmental formative evaluation, the study team will also conduct evidence-based quality improvement (EBQI), reviewing local data with stakeholders to elicit further input. For fidelity analyses, all DBT-SG sessions will be recorded and up to 15% will be analyzed for fidelity by a trained rater using the DBT- California Competence Scale – Skills Training Group, a practical, expert- developed tool for assessing DBT skills group competence. Additional information on the implementation-focused formative evaluation is provided on p. 6 of the article.

2. **Murphy SL, Zick SM, Harris RE, et al. Self-administered acupressure for veterans with chronic back pain: Study design and methodology of a type 1 hybrid effectiveness implementation randomized controlled trial. *Contemporary Clinical Trials* 2023; 130:107232. https://doi.org/10.1016/j.cct.2023.107232**

   - **Hybrid Type 1 Goals Addressed:** Goals 1 and 2

   - **Objective:** Study aims are to: 1) determine effectiveness of a self-administered acupressure protocol at improving pain interference and secondary outcomes of fatigue, sleep quality, and disability in 300 Veterans with chronic low back pain (CLBP); and 2) evaluate implementation barriers and facilitators to scale-up acupressure utilization within the Veterans Health Administration.

   - **Design:** Hybrid Type 1 effectiveness-implementation randomized controlled trial

   - **Setting:** VA Ann Arbor Healthcare System, including the Ann Arbor VA Medical Center and affiliated community-based outpatient clinics (CBOCs; i.e., Flint, MI, and Toledo, OH)

   - **Intervention:** Participants randomized to the intervention will be asked to follow a 6-week protocol that involves performing acupressure once daily (self-administered) guided by the MeTime app. After 6 weeks, they will be asked to stop acupressure for 4 weeks so persisting effects can be determined. All intervention participants will receive a Kindle and plastic Acuwand to apply acupressure. Participants will also be instructed that acupressure may be applied with a finger or pencil eraser. Additional information on the intervention and waitlist control conditions is provided on pp. 3-4 of the article.

- **Summary of Hybrid Type 1 Study Methodology**
  Effectiveness: Authors used four main sources to guide selection of effectiveness measures: International Consensus Group, Initiative on Methods, Measurement, and Pain Assessment in Clinical Trials (which recommends core outcome measures for chronic pain trials); NIH Task Force on Research Standards for Chronic Low Back Pain; VA Pain Measurement Outcomes Workgroup; and their own pilot clinical trial. Table 1 on p. 5 of the article lists the effectiveness measures and data collection timeline.

  Implementation: Implementation-focused data will be collected through semi-structured interviews with providers and collaborators, a provider survey, a patient survey, and other process data collected throughout the study. This includes documentation of the number of referrals, number of classes offered, and fidelity assessments to ensure appropriate delivery of course content and acupressure use by intervention participants. The implementation evaluation will primarily be guided by the RE-AIM model, which guides selection of measures according to five domains: Reach, Effectiveness (see above), Adoption, Implementation, and Maintenance. The 'Aim 2 analysis' section on p. 6 of the article provides additional information on RE-AIM measures to be collected and plans for data analysis.

Non-VA Exemplars

3. **Edelman EJ, Dziura J, Deng Y, et al. A SMARTTT approach to Treating Tobacco use disorder in persons with HIV (SMARTTT): Rationale and design for a hybrid type 1 effectiveness-implementation study. *Contemporary Clinical Trials* 2021, 110:106379. https://doi.org/10.1016/j.cct.2021.106379**

   - **Hybrid Type 1 Goals Addressed:** Goals 1 and 2

   - **Objective:** To describe the rationale, aims, and design for a Hybrid Type 1 study to test a clinical intervention to treat tobacco use disorder in persons with HIV (PWH). Overall study goal is to identify the optimal adaptive treatment strategy to promote smoking abstinence in PWH who smoke cigarettes.

   - **Setting:** The 3 participating health systems include: Yale New Haven Hospital (YNHH)'s Nathan Smith Clinic and Haelen Center, New Haven, CT; SUNY Downstate's STAR Health Center, Brooklyn, NY; and Mount Sinai's Institute for Advanced Medicine's Jack Martin Clinic and Morningside Clinic, New York, NY.

   - **Design:** Sequential multiple assignment randomized trial (SMART).

   - **Intervention:** Nicotine replacement therapy (NRT) with or without contingency management (NRT vs. NRT + CM). Participants with response (defined as exhaled carbon monoxide (eCO)-confirmed smoking abstinence at week 12) continue the same treatment for another 12 weeks. Participants with non-response are re-randomized to either switch medications from NRT to varenicline or intensify treatment to a higher CM reward schedule. Interventions are delivered by clinical pharmacists embedded in HIV clinics.

- **Summary of Hybrid Type 1 Study Methodology**
  Effectiveness: Primary effectiveness outcome is self-reported past 7-day smoking abstinence confirmed by exhaled carbon monoxide (eCO). Secondary effectiveness outcomes include CD4 cell count, HIV viral load suppression, and Veterans Aging Cohort Study (VACS) Index 2.0 scores. Additional detail on trial design and methods for evaluating effectiveness are provided in Sections 2.1 - 2.10.2.3 on pp. 2-6 of the article.

  Implementation: The study plans to conduct a complementary implementation-focused process evaluation to assess feasibility, cost, and future implementation of the clinical intervention (see Section 2.11 on p. 6 of the paper). The process evaluation is grounded in the *Reach, Effectiveness, Adoption, Implementation, and Maintenance* (*RE-AIM*) and *Promoting Action on Research Implementation in Health Services* (*PARiHS*) implementation science frameworks. Table 3 (page 7 of the article) provides an overview of the implementation-focused process evaluation— including questions, data sources, and tools to be used, organized by elements of the RE-AIM and PARIHS frameworks. Appendix 2 of the article includes an online survey utilized to examine participants' pre/post perspectives on the clinical intervention package and its components among clinicians, staff, and clinical leadership at participating sites. Finally, to assess costs associated with the clinical intervention, the authors describe plans to track clinical pharmacist time for training and then delivering the intervention, costs of eCO monitoring, and rewards earned per CM session. The authors also describe plans to track costs from the participants' perspective as well as changes in health service utilization and quality of life for future cost-effectiveness analyses.

4. **Young AM, Havens JR, Cooper HLF, et al. Kentucky Outreach Service Kiosk (KyOSK) Study protocol: a community-level, controlled quasi-experimental, type 1 hybrid effectiveness study to assess implementation, effectiveness and cost-effectiveness of a community-tailored harm reduction kiosk on HIV, HCV and overdose risk in rural Appalachia. *BMJ Open* 2024; 14(3):e083983. https://doi.org/10.1136/bmjopen-2024-083983**

   - **Hybrid Type 1 Goals Addressed:** Goals 1 and 2

   - **Objective:** To describe the Kentucky Outreach Service Kiosk (KyOSK) Study protocol to test the effectiveness, implementation outcomes and cost-effectiveness of a community-tailored, harm reduction kiosk in reducing HIV, hepatitis C and overdose risk in rural Appalachia.

   - **Setting:** Two rural Appalachian Kentucky counties similar in demographic and epidemiological profile, both having been designated as 'distressed' or 'at-risk' based on economic indicators.

   - **Design:** Community-level, controlled quasi-experimental, non-randomized trial; the study will enroll 750 people who use drugs (PWUDs), 425 in the intervention county and 325 in the control county.

   - **Intervention:** A syringe service program (SSP) staffed by the local health department in the control county serves as the standard-of-care comparison. The SSP provides syringes, cookers, cottons, naloxone, fentanyl test strips, wound care kits, condoms/lubricant, snacks, drinks and sharps containers. SSP clients have similar access to harm reduction supplies as those accessing the kiosk in the intervention county (see below) and will receive a trifold resource guide with

information on services and contact information for recovery coaches. ***In the intervention county***, an existing SSP will be enhanced with a kiosk stocked with harm reduction supplies, including a sharps receptacle equipped with technology to approximate the number of returned syringes and determine the number allowed to be dispensed. The kiosk will also feature a care navigation call-back menu. Participants will select services displayed on the kiosk's interface (see table 3 on p. 4 of the article for potential menu) and provide their phone number(s) for call-back. People with lived experience with substance use who are certified and trained recovery coaches (RCs) will monitor the kiosk data dashboard and field call-back requests within 3 business days. RCs will briefly assess service needs and potential barriers and make facilitated referrals to health and support services.

- **Summary of Hybrid Type 1 Study Methodology**
  Effectiveness: Primary and secondary effectiveness outcomes are listed and defined in Table 4 on p. 5 of the article. The study will develop and calibrate a deterministic model of hepatitis C virus (HCV) transmission and overdose among PWUD in the intervention county to estimate the kiosk's impact and cost-effectiveness. Impact will be measured as reductions in HCV incidence/prevalence, HCV infections and overdoses averted, and quality-adjusted life-years (QALYs) saved over the study and longer time frames (10/20/50 years).

  Implementation: Following the *Implementation Outcomes Framework*, the study will assess acceptability, appropriateness, fidelity, cost, penetration/reach and sustainability. Acceptability and appropriateness will be assessed in surveys using the Acceptability of Implementation Measure and Intervention Appropriateness Measure, respectively. Fidelity of kiosk and staffed SSP implementation will be assessed early and mid-trial on three domains: (1) supply availability, (2) operation and (3) recovery coaching (see p. 6 of the article for more information on fidelity assessment). Costs will be estimated from the provider's perspective using a micro-costing approach that measures and values in monetary terms all resources invested and links costs to the primary and secondary effectiveness outcomes to evaluate economic impact. Penetration (reach) will be determined by examining the number who engage with the kiosk and/or staffed SSP divided by the number enrolled at the time of intervention/comparison condition implementation (i.e., percent who use the kiosk or SSP) and per supply (i.e., percent who accessed each supply) at monthly intervals. Prospects for sustainment will be explored in the final year using qualitative, semi-structured interviews with SSP and other health department staff and local and state leadership.

5. **Hassan S, Sobers N, Paul-Charles J, et al. Diabetes prevention in the Caribbean using Lifestyle Intervention and Metformin Escalation (LIME): Protocol for a hybrid Type-1 effectiveness-implementation trial using a quasi-experimental study design. *Contemporary Clinical Trials Communications* 2021, *22*:100750. https://doi.org/10.1016/j.conctc.2021.100750**

- **Hybrid Type 1 Goals Addressed:** Goals 1 and 2

- **Objective:** The objective of this study is to test the hypothesis that a tailored lifestyle modification program for Caribbean-descent populations (Lifestyle Intervention and Metformin Escalation (LIME)) combined with guideline compliant medication prescribing of metformin will reduce hemoglobin A1c in high-risk individuals with prediabetes. This paper presents the rationale for the LIME intervention and the Hybrid Type 1 study protocol to assess its

effectiveness and implementation.

- **Design:** Quasi-experimental, pre-post study design with a non-equivalent control group.

- **Setting:** Four clinical sites in Barbados (St. Michael), Puerto Rico (Carolina), Trinidad (South West Region), and the US Virgin Islands (St. Thomas).

- **Intervention:** The LIME lifestyle modification program is based on the NIH/NIMHD-funded Project HEED (Help Educate to Eliminate Diabetes), a diabetes prevention lifestyle curriculum that used a community-based participatory research approach to modify the Chronic Disease Self-Management Program to diabetes prevention. HEED was culturally adapted to the Caribbean context to create the LIME workshop curriculum. LIME workshops (taught by two certified workshop leaders) entail six weekly 2.5-h sessions focused on principles of self-efficacy, teaching healthy eating habits, and increasing physical activity. Workshop leaders called participants 3 months after the start of the workshops to discuss the extent to which they were continuing lifestyle changes. Additional information on the LIME program is provided on pp. 3-4 of the article.

- **Summary of Hybrid Type 1 Study Methodology**
  Effectiveness: The primary effectiveness outcome for the study is change in HbA1c. Secondary outcomes include changes in weight, other anthropometric measures of obesity (waist circumference, waist-to-hip ratio, BMI), cholesterol, blood pressure, diabetes self-efficacy score, and quality of life.

  Implementation: For the study objective focused on implementation, the RE-AIM model was used to guide selection of the outcomes of interest. Specifically, implementation outcomes include measures of reach, adoption (individual/institutional), implementation (fidelity, acceptability, appropriateness, and cost), and maintenance (sustainability). Effectiveness outcomes (i.e., the 'E' in RE-AIM) are described above. Additional information on the study's evaluation plan is provided on pp. 4-5 of the article, including further specification of 'Reach', 'Effectiveness', 'Adoption', 'Implementation', and 'Maintenance' outcome measures provided in Table 2 on p. 5.

Other Hybrid Type 1 Study Examples

Beisheim-Ryan EH, Butera KA, Hinrichs LA, Derlein DL, Malone DJ, Holtrop JS, Forster JE, Diedrich D, Gustavson AM, Stevens-Lapsley JE. Advancing Rehabilitation Paradigms for Older Adults in Skilled Nursing Facilities: An Effectiveness-Implementation Hybrid Type 1 Clinical Trial Protocol. Phys Ther. 2023 Sep 1;103(9):pzad053. doi: 10.1093/ptj/pzad053.

Breland JY, Fletcher TL, Maguen S, Timko C, Raikov I, Boothroyd DB, Frayne SM. The EMBER trial for weight management engagement: A hybrid type 1 randomized controlled trial protocol. Contemp Clin Trials. 2023 Dec;135:107364. doi: 10.1016/j.cct.2023.107364.

Callegari LS, Benson SK, Mahorter SS, Nelson KM, Arterburn DE, Hamilton AB, Taylor L, Hunter-Merrill R, Gawron LM, Dehlendorf C, Borrero S. Evaluating the MyPath web-based reproductive decision support tool in VA primary care: Protocol for a pragmatic cluster randomized trial. Contemp Clin Trials. 2022 Nov;122:106940. doi: 10.1016/j.cct.2022.106940.

Donovan LM, McDowell JA, Pannick AP, Pai J, Bais AF, Plumley R, Wai TH, Grunwald GK, Josey K, Sayre GG, Helfrich CD, Zeliadt SB, Hoerster KD, Ma J, Au DH. Protocol for a pragmatic trial testing a self-directed lifestyle program targeting weight loss among patients with obstructive sleep apnea (POWER Trial). Contemp Clin Trials. 2023 Dec;135:107378. doi: 10.1016/j.cct.2023.107378.

Edelman EJ, Rojas-Perez OF, Nich C, Corvino J, Frankforter T, Gordon D, Jordan A, Paris M Jr, Weimer MB, Yates BT, Williams EC, Kiluk BD. Promoting alcohol treatment engagement post-hospitalization with brief intervention, medications and CBT4CBT: protocol for a randomized clinical trial in a diverse patient population. Addict Sci Clin Pract. 2023 Sep 19;18(1):55. doi: 10.1186/s13722-023-00407-9.

Gladstone, T. R. G., Feinstein, R. T., Fitzgibbon, M. L., Schiffer, L., Berbaum, M. L., Lefaiver, C., Pössel, P., Diviak, K., Wang, T., Knepper, A. K., Sanchez-Flack, J., Rusiewski, C., Potts, D., Buchholz, K. R., Myers, T. L., & Van Voorhees, B. W. (2022). PATH 2 Purpose: Design of a comparative effectiveness study of prevention programs for adolescents at-risk for depression in the primary care setting. *Contemporary clinical trials*, *117*, 106763. https://doi.org/10.1016/j.cct.2022.106763

Hadlandsmyth K, Burgess DJ, Leparski RF, Odom AS, Campbell EJ, Obrecht AA, Adamowicz JL, Cho H, Steffensmeier KS, Johnson NL, Richards CC, Vander Weg MW, Lund BC, Yoon P, Mosher HJ. The Perioperative Pain Self-Management (PePS) randomized controlled trial protocol: Preventing chronic post-surgical pain and prolonged opioid use. Contemp Clin Trials. 2022 Jul;118:106810. doi: 10.1016/j.cct.2022.106810.

Hurlocker MC, Moyers TB, Hatch M, Curran G, McCrady B, Venner KL, Witkiewitz K. Effectiveness and feasibility of a motivational interviewing intake (MII) intervention for increasing client engagement in outpatient addiction treatment: an effectiveness-implementation hybrid design protocol. Addict Sci Clin Pract. 2023 Oct 21;18(1):63. doi: 10.1186/s13722-023-00412-y.

Lin, E., Uhler, L. M., Finley, E. P., Jayakumar, P., Rathouz, P. J., Bozic, K. J., & Tsevat, J. (2022). Incorporating patient-reported outcomes into shared decision-making in the management of patients with osteoarthritis of the knee: a hybrid effectiveness-implementation study protocol. *BMJ open*, *12*(2), e055933. https://doi.org/10.1136/bmjopen-2021-055933

Mayberry LS, Felix HC, Hudson J, Curran GM, Long CR, Selig JP, Carleton A, Baig A, Warshaw H, Peyrot M, McElfish PA. Effectiveness-implementation trial comparing a family model of diabetes self-management education and support with a standard model. Contemp Clin Trials. 2022 Oct;121:106921. doi: 10.1016/j.cct.2022.106921.

Park, L. S., Chih, M. Y., Stephenson, C., Schumacher, N., Brown, R., Gustafson, D., Barrett, B., & Quanbeck, A. (2022). Testing an mHealth System for Individuals With Mild to Moderate Alcohol Use Disorders: Protocol for a Type 1 Hybrid Effectiveness-Implementation Trial. *JMIR research protocols*, *11*(2), e31109. https://doi.org/10.2196/31109

Pisani A, Connor K, Van Orden K, Jordan N, Landes S, Curran G, McDermott M, Ertefaie A, Kelberman C, Ramanathan S, Carruthers J, Mossgraber K, Goldston D. Effectiveness of a targeted brief intervention for recent suicide attempt survivors: a randomised controlled trial protocol. BMJ Open. 2023 Mar 3;13(3):e070105. doi: 10.1136/bmjopen-2022-070105.

Reger MA, Lauver MG, Manchester C, Abraham TH, Landes SJ, Garrido MM, Griffin C, Woods JA, Strombotne KL, Hughes G. Development of the Veterans Crisis Line Caring Letters Suicide Prevention Intervention. Health Serv Res. 2022 Jun;57 Suppl 1(Suppl 1):42-52. doi: 10.1111/1475-6773.13985.

Ricke, E., Dijkstra, A., & Bakker, E. W. (2023). Feasibility, effectiveness and safety of self-management in pulmonary rehabilitation: a study protocol using a hybrid type 1 effectiveness-implementation design. *Frontiers in rehabilitation sciences*, *4*, 1178823. https://doi.org/10.3389/fresc.2023.1178823

Schapira MM, Chhatre S, Prigge JM, Meline J, Kaminstein D, Rodriguez KL, Fraenkel L, Kravetz JD, Whittle J, Bastian LA, Vachani A, Akers S, Schrand S, Ibarra JV, Asan O. A Veteran-Centric Web-Based Decision Aid for Lung Cancer Screening: Usability Analysis. JMIR Form Res. 2022 Apr 8;6(4):e29039. doi: 10.2196/29039.

Smith, S. L., Nyirandagijimana, B., Hakizimana, J., Levy, R. P., Bienvenu, R., Uwamwezi, A., Hakizimfura, O., Uwimana, E., Kundu, P., Mpanumusingo, E., Nshimyiryo, A., Rusangwa, C., Kateera, F., Mukasakindi, H., & Raviola, G. (2021). Evaluating the delivery of Problem Management Plus in primary care settings in rural Rwanda: a study protocol using a pragmatic randomised hybrid type 1 effectiveness-implementation design. *BMJ open*, *11*(12), e054630. https://doi.org/10.1136/bmjopen-2021-054630

Stanley, B., Labouliere, C. D., Brown, G. K., Green, K. L., Galfalvy, H. C., Finnerty, M. T., Vasan, P., Cummings, A. K., Wainberg, M., Carruthers, J. W., & Dixon, L. B. (2021). Zero suicide implementation-effectiveness trial study protocol in outpatient behavioral health using the A-I-M suicide prevention model. *Contemporary clinical trials*, *100*, 106224. https://doi.org/10.1016/j.cct.2020.106224

Suresh, K., Holtrop, J. S., Dickinson, L. M., et al. (2022). PATHWEIGH, pragmatic weight management in adult patients in primary care in Colorado, USA: study protocol for a stepped wedge cluster randomized trial. *Trials*, *23*(1), 26. https://doi.org/10.1186/s13063-021-05954-7
